# Different delayed consequences of attaining a plateau phase in practicing a simple (finger-tapping sequence learning) and a complex (Tower of Hanoi puzzle) task

**DOI:** 10.3758/s13421-024-01622-8

**Published:** 2024-09-03

**Authors:** Iris Lowenscuss-Erlich, Avi Karni, Carmit Gal, Eli Vakil

**Affiliations:** 1 Department of Psychology and Leslie and Susan Gonda (Goldschmied) Multidisciplinary Brain Research Center, Bar-Ilan University, 52900 Ramat-Gan, Israel; 2 Sagol Department of Neurobiology and the E.J. Safra Brain Research Center for the Study of Learning Disabilities, University of Haifa, Haifa, Israel

**Keywords:** Skill learning, Finger tapping sequence, Tower of Hanoi puzzle, Plateau phase

## Abstract

In practicing a new task, the initial performance gains, across consecutive trials, decrease; in the following phase, performance tends to plateau. However, after a long delay additional performance improvements may emerge (delayed/ “offline” gains). It has been suggested that the attainment of the plateau phase is a necessary condition for the triggering of skill consolidation processes that lead to the expression of delayed gains. Here we compared the effect of a long-delay (24–48 h) interval following each of the two within-session phases, on performance in a simple motor task, the finger-tapping sequence learning (FTSL), and in a conceptually complex task, the Tower of Hanoi puzzle (TOHP). In Experiment 1 we determined the amount of practice leading to the plateau phase within a single practice session (long practice), in each task. Experiment 2 consisted of three consecutive sessions with long-delay intervals in between; in the first session, participants underwent a short practice without attaining the plateau phase, but in the next two sessions, participants received long practice, attaining the plateau phase. In the FTSL, short practice resulted in no delayed gains after the long delay, but after 24–48 h following long practice, task performance was further improved. In contrast, no delayed gains evolved in the TOHP during the 24- to 48-h delay following long practice. We propose that the attainment of a plateau phase can indicate either the attainment of a comprehensive task solution routine (achievable for simple tasks) or a preservation of work-in-progress task solution routine (complex tasks); performance after a long post-practice interval can differentiate these two states.

## Introduction

Unlike declarative memory ("what-where-who," statements and episodic occurrences), which may be generated even when only a single event is experienced, the triggering of procedural memory (“what-how to do,” skills, habits, and recurring patterns in experience) necessitates multiple repetitions (practice). Across these multiple repetitions task performance improves in the form of a power function with large gains across the initial task iterations and subsequently reaching a plateau phase wherein further practice leads too little or no appartent gains (Hauptmann & Karni, 2002; Korman et al., 2003). Importantly, this pattern is charecteristic of learning a given task in multiple domains – perceptual (e.g., Karni, 1996; Karni & Bertini, 1997), motor (e.g., Hauptmann & Karni, 2002; Hauptmann et al., 2005), and cognitive (e.g., Fox et al., 2017). During the hours-long interval that follows a practice session, the gains in performance attained in the practice session can be stabilized, i.e., the gains accrued in the practice session are maintained and can become immune to potential interference by subsequent experiences (Brashers-Krug et al., 1996; Korman et al., 2007; Maaravi Hesseg et al., 2016; Robertson et al., 2004a, 2004b; Stickgold & Walker, 2005). However, after some training protocols, significant gains in the performance of the practiced task are noted after practice has terminated (delayed, “offline” gains). These delayed gains do not appear immediately after training, but, rather, after a delay period wherein performance remains unchanged compared to the performance attained at the end of the practice session; this “latent” interval can take, in young adults, from a few hours to a day or two (Karni & Bertini, 1997; Karni & Sagi, 1993; Korman et al., 2003; Luft & Buitrago, 2005; Maquet et al., 2003; Robertson et al., 2004a, 2004b; Song, 2009; Stickgold & Walker, 2005; Wilhelm et al., 2012). Whether the expression of delayed gains is subserved by the same mechanisms subserving memory stabilization is not clear. There is evidence suggesting that the conditions for long-term stabilization (and retention) and the expression (and retention) of delayed gains, may differ (e.g., Adi-Japha & Karni, 2016; Korman et al., 2007; Robertson et al., 2004a, 2004b; Walker, 2005), but there are also indications that the two mnemonic expressions are expressions of a shared core process (Dudai, 2012; Korman et al., 2007; Maaravi Hesseg et al., 2016).

Stabilization and delayed gains in performance presumably reflect long-lasting experience-dependent changes within the neural networks used for task performance (Dudai et al., 2015; Gabitov et al., 2014; Squire, 2004). There is good evidence suggesting that such practice-dependent changes involve structural changes at the synaptic level, a process that takes time to be completed (e.g., Rogerson et al., 2014; Xu, YU, et al., 2009; Yang et al., 2009, 2014). When completed, the structural changes resulting from these processes can account for the finding of both long-term stabilization and the emergence of “delayed” gains (Karni, 1996).

In laboratory settings, skill acquisition (for the execution of a specific novel task) can be characterized by two distinct phases that occur within the early training sessions, especially with the first encounter with the new task: an initial, *fast* phase of performance improvement, and subsequently a slowing in the rate of improvement and as practice continues, the attainment of stable performance (*plateau* phase) (e.g., Hauptmann & Karni, 2002; Karni & Sagi, 1993; Korman et al., 2003; Yang et al., 2021). These two phases of performance improvement, within the training session, are, however, characteristic of learning only in the early sessions of multi-session training protocols; as practice continues in multi-session training protocols, the within-session gains tend to diminish (e.g., Hauptmann & Karni, 2002; Karni & Sagi, 1993; Korman et al., 2003, 2018). Typically, the fast phase of improvement is very short in later sessions (apparent across only one or a few trials; often considered as warm-up or re-activation trials; Hauptmann & Karni, 2002; Korman et al., 2003), and performance tends to be stable within the sessions.

Evidence that skill is acquired in phases, even within a single session, comes from studies of perceptual, motor, and cognitive task learning (Anderson, 1982; Fitts, 1964; Fitts & Posner, 1967; Hauptmann & Karni, 2002; Hauptmann & Karni, 2002; Karni & Sagi, 1993; Karni et al., 1995; Willingham, 1998). Central to many (e.g., Anderson, 1982; Anderson et al., 2004; Fitts, 1964; Fitts & Posner, 1967) conceptual frameworks is the notion of an increasing (not necessarily explicit) grasp of the task requirements, an increased reliance on aspects and components that are relevant for improved task performance, and then the integration of these elements into a task-specific, comprehensive solution routine. The learner establishes performance routines through a selection and an assemblage of existing processing modules that can be effective for task solution (*knowledge compilation* in Anderson’s terminology; Anderson, 1982; Anderson et al., 2004)*.* This can lead to a select, new, task procedure that can undergo consolidation, stabilization, and enhancement, as part of a *proceduralization or automatization process* (e.g., Anderson et al., 2004, and Fitts, 1964, respectively). The new task-solution procedure (routine, skill) can be maintained, if consolidated, as the basis for a durable and fluent “what and how to do” knowledge.

Although the process of establishing durable fluent “what to do” and “how to do” knowledge has been followed in many simple motor and perceptual tasks, in laboratory settings, across multiple time-points, the process of skill mastery has not been sufficiently established in more complex tasks, especially in tasks that are conceptually complex and demanding. There is good evidence showing that as in motor and perceptual tasks a fast phase of learning followed by a plateau phase characterize the learning of quite complex cognitive tasks. Post-session stabilization has been noted following extensive training on complex conceptual tasks, for example in the TOHP (Vakil & Hoffman, 2004; Vakil et al., 2014). However, it is not clear whether a distinct post-session consolidation phase follows practice on such tasks if a plateau phase is attained, as may occur in the practice of simpler motor and perceptual tasks. There is evidence suggesting that the initiation of consolidation processes that result in delayed gains is dependent on the affordance of sufficient practice and the attainment of a plateau phase (e.g., Hauptmann & Karni, 2002; Hauptmann et al., 2005). It is not known if and under what conditions delayed gains in performance can emerge in the post-session interval after practice on such tasks.

In attempting to directly address the question of whether delayed gains in performance can also emerge in the post-session interval after practice on cognitive tasks, one must acknowledge the difficulty inherent in the multiple differences (e.g., level of complexity; attention demands; involvement of strategy choice or explicit knowledge) between cognitive and “simpler” perceptual or motor tasks. These differences, one must assume, would be reflected even in, for example, different quantitative requirements for training (amount of practice) to attain different phases in learning and the induction of memory processes. Our approach was based on the assumption that the attainment of a plateau by the end of a training session can serve as an operational criterion for the attainment of a comparable level of skill (phase of learning) in different tasks (Adi-Japha & Karni, 2016; Chiharu et al., 2019). Thus, the notion underlying our experiments was that despite the large differences between tasks, in multiple aspects and parameters, all tasks in which skill can be attained share, in the process of practice, a fundamental time-course of gaining expertise and specifically undergo learning characterized by the same distinct, fast, and slow phases.

Therefore, to enable a comparison across two different tasks our approach was to ensure that trainees practiced the two tasks to the point where their performance (specifically in terms of trial completion time, but with no tradeoff with accuracy) attained a plateau (Experiments 1a and 1b).

In a previous study (Vakil et al., 2014) we have shown that performance gains attained in a practice session on the TOHP can be well retained across a delay interval of 24–48 h, suggesting a stabilization of the performance gains (although some fast learning did occur at the beginning of the second session, after the delay interval). However, we did not systematically control for the attainment of a plateau phase in performance prior to the affordance of a delay interval and we did not address the possibility of a stabilization following short training in the TOHP. In the current study, we addressed both these questions (Experiment 2) and compared, within the same group of participants, the effects of a delay interval (24 h long) following short (brief) training and the effect of a comparable delay interval after participants were afforded training sufficient to attain a plateau phase in both the TOHP and in the FTSL task.

The aim of Experiments 1a and 1b was therefore to determine how much training is required to go through the fast phase and reach the slow phase of learning within a single practice session, in the two tasks, FTSL and TOHP. The fast phase of learning, as reported above, is characterized by significant improvements in performance from one block to another, while in the slow phase the improvement from one block to the next will not be significant.

## Experiment 1a

### Motor task – finger-tapping sequence learning (FTSL)

#### Methods

**Participants.** Participants (*n* = 20, 15 females) were undergraduate students who took part in the experiment to fulfill academic requirements (age: 19–26 years). Performance of both these tasks stabilizes at the end of the second decade in typical development and similar levels of performance are maintained throughout adulthood (Diamond, 2006; Dorfberger et al., 2007). Three participants were excluded due to repeated presses of single keys (without being aware) resulting in a record of multiple repetitive key presses. Given that the task required the performance of a specified sequence, these participants’ data were unusable/informative. The study was approved by the Institutional Review Board at Bar-Ilan University. Informed consent was obtained from all participants.

**Task and procedure.** The FTSL task used in the current study is a computerized version of the initial FOS task (Karni et al., 1995). The study was run in a well-lit (office room-lighting conditions) quiet laboratory setting. The version is the FTSL task that was programed on SuperLab software, which also requires key presses on a specially adapted keyboard with four keys arranged with numbers 1 to 4 that records performance time in milliseconds. The task consisted of repeating (tapping) a sequence of five movements 4, 1, 3, 2, 3, as quickly and accurately as possible, using the dominant hand. Each participant sat in front of a computer screen ((size 42 × 4 cm; resolution 1,600 × 900 pixels) positioned about 60 cm from the viewer), the dominant hand was placed on the keyboard so that each finger was placed, comfortably, on a different matching key 1 to 4.

All participants were trained in a 15-min session. Full explicit knowledge and demonstration of the required movement sequence was provided. Performance training was initiated only after participants were able to correctly generate three sequences consecutively, without visual feedback, as an indication that the participant understood and was able to generate the required key-press sequence. The training session consisted of 15 blocks, consisting of ten sequence repetitions (i.e., a total of 50 key-presses per block). Each block was interspersed with a mandatory, minimum 60-s rest period, but a break of more than 3 min was not allowed. At the beginning of each block all participants were instructed to continuously tap the sequence “as quickly and accurately as possible,” using their dominant hand, immediately after seeing a visual “go” cue; at the end of each block, a visual “stop” cue appeared. Participants were instructed that occasional errors should not be corrected and were required to continue the task without pause. No feedback on any performance measure was provided. The response times of key presses were automatically recorded. For the analyses of the results, for each participant we calculated the correctly executed sequences per block (0–10), and for every correct sequence the median was generated. For each block the mean of the medians was computed and was used for all data analyses as the block score.

## Results and discussion

**Fig. 1:**
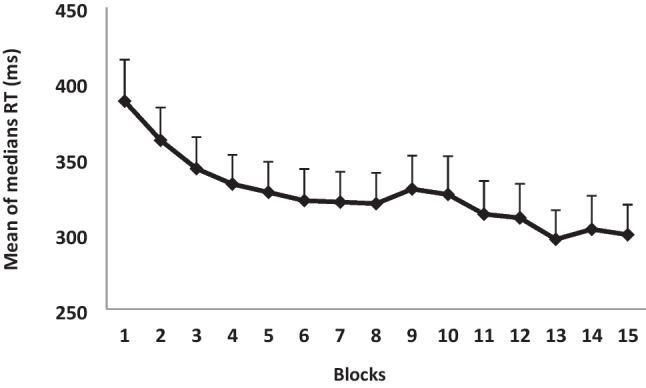
The mean of medians of correct sequences in each one of the 15 blocks, in the computerized sequential finger-tapping task. Bars = *SE*

The mean of medians of response time (RT; speed) in each of the 15 blocks was calculated and used as a measure for comparing performance changes within the practice session. A repeated-measures generalized linear model (GLM) analysis on the mean of median RT, as a within-subjects factor, showed that there was a significant effect for training *F*(14, 224) = 9.59, *p* < 0.001, ${\eta }_{p}^{2}$ = 0.37. Throughout the session, accuracy, i.e., percent correct responses, ranged from 95 to 100%. Thus, there was no speed–accuracy trade-off. A follow-up contrast analysis showed that the first three blocks were significantly different from each other (*p* < 0.05), and from the fourth block on differences between successive blocks were not significant. Therefore, the first three blocks, when participants were still in the first phase of learning, were defined as the “fast” phase; the “plateau” phase consisted of the fourth to the 15th blocks (Fig. 1).

Because differences between the training blocks were variable across participants, we enlarged the number of training blocks to 20 in Experiment 2a to ensure that participants’ performance stabilized, i.e., that all participants were likely to experience the second “plateau” phase, by the latter part of the practice session.

## Experiment 1b

### Cognitive task – Tower of Hanoi puzzle (TOHP)

#### Methods

**Participants. **Participants (*n* = 26, 22 females) were undergraduate students who took part in the experiment to fulfill academic requirements. Ages ranged from 19 to 30 years (*M* = 22.6 years). Two participants were excluded due to very slow performance (2 *SD* below the mean) or very fast performance (participants who might have been exposed to the task). Informed consent was obtained from all participants.

**Task and procedure.** A computerized version of the TOHP task was used. Three pegs appeared on a computer screen, numbered 1–3. Four disks were arranged according to size with the largest disk at the bottom of the extreme left peg (#1). Disk transfers were performed by tapping on the numbers 1, 2, 3 on the keyboard, which corresponded to the three pegs. Participants were told that the goal was to move the disks from the left-most peg (#1) to the right-most peg (#3) in a minimum number of moves, and to do it as fast as possible. In addition, they had to abide by the following rules: only one disk could be moved at a time; no disk could be placed on a smaller one; the middle peg had to be used. The optimal solution for four disks requires 15 moves. The average time per move and the number of moves required to solve the puzzle were recorded for the analysis. Participants were tested individually; training was held over one session, for approximately 15 min, and participants were required to solve the TOHP consecutively in 18 trials, in the minimum number of moves and as quickly as possible. After every successful trial, there was an “enter” sign to proceed to the next trial.

#### ***Results and discussion***

**Fig. 2:**
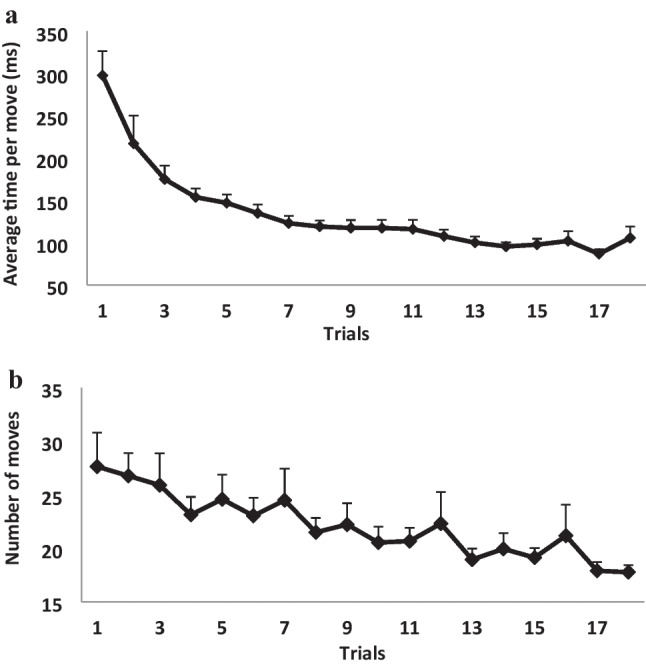
(**A**) The average time per move (s) in the 18 trials, in the Tower of Hanoi puzzle (TOHP). Bars = *SE*. (**B**) The number of moves in every trial, until reaching the solution in the TOHP

Two dependent measures were analyzed separately: average time per move and number of moves required for solving a TOHP problem in 18 trials. We did not use the total time for trial completion as a measure of performance speed (performance time) because this measure is confounded by the number of moves. The results are presented in Figs. 2a and 2b. A repeated-measures GLM analysis on 18 successive trials as within-subjects factors showed that both the average time per move and the mean number of moves required for solving the TOHP problem significantly improved through the session, *F*(17, 357) = 19.48, *p* < 0.001, ${\eta }_{p}^{2}$ = 0.48; *F*(17, 357) = 2.84, *p* < 0.001, ${\eta }_{p}^{2}$ = 0.12, respectively. A follow-up contrast analysis showed that this significant learning effect, for the average time per move measure, reflected significant differences between the first three successive trials (*p* < 0.05), but from the fourth trial on, the differences were not consistent. For the number of moves, improvement was very gradual, so we did not find a clear indication for demarcating a fast and slow phase of learning. Thus, the first phase of training was determined solely on the average time per moves measure. The first three blocks were defined as the first, fast phase of learning, and the second, slow phase consisted of the 15 trials starting from the fourth block and on.

Based on the results of Experiments 1a and 1b, we designed Experiment 2, the main experiment, to address the effects of 24- to 48-h long delays after short and then long practice in the motor FTSL and the cognitively more demanding TOHP, on subsequent performance (Experiments 2a and 2b, respectively).

## Experiment 2a

### Motor task – FTSL

#### Methods

**Participants.** Participants (*n* = 25, 21 females) were undergraduate students at Bar-Ilan University (Israel) who took part in the experiment to fulfill academic requirements. Ages ranged from 20 to 27 years) (*M* = 21.2 years). Five participants were excluded due to incorrect tapping on one key (for example 4, 1, 1, 3, 2, 3), without being aware of it. Since the length of their training was shorter than that of other participants, their results were not comparable to the other participants. Informed consent was obtained from all participants.

### Task and procedure

#### The FTSL task

The FTSL task was run using the protocol described in Experiment 1a.

**Fig. 3:**
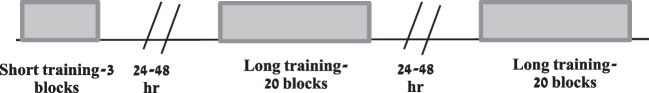
The timeline and design of Experiment 2: three training sessions, 24–48 h apart. The first session included three blocks of training, second and third included 20 blocks (*n* = 25)

The experiment included three sessions, 24–48 h apart. In the first session, participants underwent a short training, consisting of three blocks of ten sequences (as described in Experiment 1). In the second and third sessions, participants underwent long training, including 20 blocks of ten sequences (Fig. 3)

#### Results

Accuracy, the number of correct sequences, was maintained high throughout the study (90–100%). Thus, as in Experiment 1a accuracy, there was no speed–accuracy trade-off. The analysis therefore focused on the RT (speed) data.

Three analyses were conducted, to test the performance changes within and between the three sessions using repeated-measures GLM to compare the mean of median RTs for correct sequences in each block. (1) Short training effect: The effect of short training was tested by comparing the three learning blocks of the first session to the first three blocks of the second and third sessions. (2) Long training effect: Performance in the long training afforded in the second session was compared to performance in the third session (20 blocks in each session). (3) Long training effect: The delay effect of 24–48 h after short training (the last block of the first session to the first block of the second session) was compared to the delay effect after long training (the final block of the second session with the first block of the third session).

### Short training effect

**Fig. 4:**
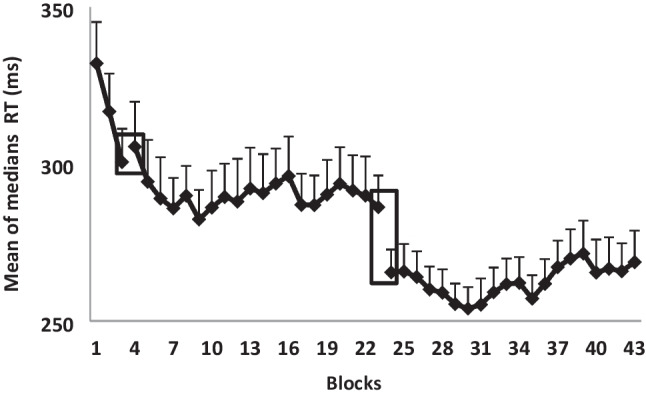
The mean of medians of correct sequences in each one of the performance blocks in the three sessions: session 1 (short training: blocks 1–3) and long training: session 2 (blocks 4–23) and session 3 (blocks 24–43), blocks, in the computerized sequential finger-tapping task. Bars = *SE*

The results are presented in Fig. 4. There was a significant learning effect, *F*(2, 38) = 8.14, *p* < 0.05, ${\eta }_{p}^{2}$ = 0.30. There was also a significant session effect, *F*(2, 38) = 25.93, *p* < 0.001, ${\eta }_{p}^{2}$ = 0.58. In addition, there was a significant interaction between learning trials and session, *F*(4, 76) = 3.56, *p* < 0.01, ${\eta }_{p}^{2}$ = 0.158, indicating that the learning rate in the first three blocks of the three sessions was different. As can be seen in Fig. 4, in the first session, the increase in performance speed was significant and steep. In session 2, the increase in speed continued moderately; however, the between-blocks differences were not significant. In session 3, performance across the first blocks of the session completely stabilized.

### Long training effect

The results presented in Fig. 4 show a significant difference between the two sessions, *F*(1, 19) = 37.46, *p* < 0.001, ${\eta }_{p}^{2}$ = 0.66, where performance is faster in the third session than in the second session. There was no significant learning effect within each session, *F*(19, 361) = 1.23, *p* = 0.232, ${\eta }_{p}^{2}$ = 0.06, nor any session and blocks interaction, *F*(19, 361) = 0.94, *p* = 0.53, ${\eta }_{p}^{2}$ = 0.047.

### Delay effect

**Fig. 5:**
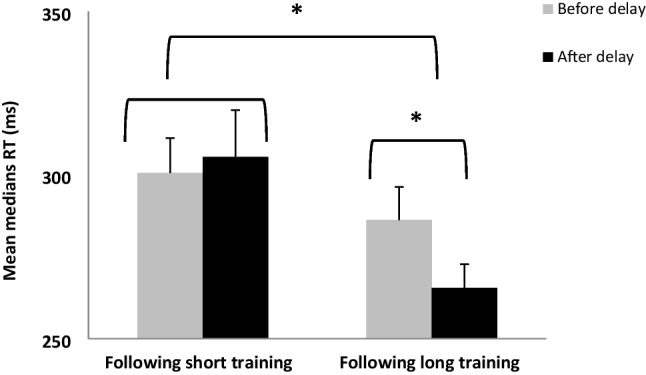
Comparison of the last block before the delay (light gray) to the first session after the delay (black) after a short training and also after a long training. Bars = *SE*; * *p* < .01

The enhancement effect over the delay interval was not significant, *F*(1, 19) = 3.06, *p* = 0.09, ${\eta }_{p}^{2}$ = 0.14. In addition, there was a significant difference between sessions, *F*(1, 19) = 11.31, *p* < 0.005, ${\eta }_{p}^{2}$ = 0.37, and a significant session by consolidation interaction, *F*(1, 19) = 5.7, *p* < 0.05, ${\eta }_{p}^{2}$ = 0.23. As can be seen in Fig. 5, the improvement in performance after a delay in the long training is significantly greater than improvement after a delay following a short training. Follow-up analyses using *t*-tests found that there were no significant differences between the last block in the first session and the first block of the second session, *t*(19) = -0.69, *p* = 0.50. However, there was a significant improvement in performance between the last block in the second session to the first in the third session, *t*(19) = 3.02, *p* < 0.01. The current results show that in performance after a delay afforded after short training (three blocks), participants started at the same level of performance 24–48 h later; there was neither significant improvement nor deterioration in speed and accuracy; participants seem to be able to retrieve the information that had been learned after short training. After a delay following long training, there was a significant improvement in performance gains. In conclusion, in a motor skill task, in the delay after short training participants preserved the level of performance before the delay, whereas after a delay following long training, consolidation effects are apparent as a significant improvement in terms of speed and with no costs in accuracy.

## Experiment 2b

### Cognitive task—TOHP

#### Methods

**Participants.** Participants (*n* = 18, ten females) were undergraduate students who took part in the experiment to fulfill academic requirements. Ages ranged from 19 to 26 years (*M* = 21.2 years). Three participants were excluded due to very slow performance (2 *SD* below the mean). Informed consent was obtained from all participants.

**Fig. 6:**
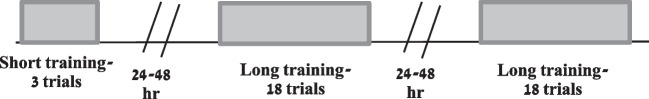
The timeline and design of Experiment 2: three training sessions, 24–48 h apart. First session included three blocks of training, second and third included 18 blocks (*n* = 18)

**Task and procedure.** The cognitive task was as described in Experiment 1b. The experiment included three sessions, 24–48 h apart. In the first session, participants underwent a short training, consisting of three trials (defined in Experiment 1b as the short training). In the second and third sessions, participants underwent long training, including 18 trials (see Fig. 6). Participants were explicitly asked not to practice either task outside of the lab sessions. This was repeated before they left the lab.

#### Results

As with the FTSL task, three analyses were conducted using a repeated-measures GLM: (1) the effect of short training, (2) the effect of long training, and (3) the effect of delay after short versus long training. The two dependent measures were analyzed separately: Average time per move and number of moves (as described in Experiment 1b).

#### *Short training effect* – *average time per moves*

**Fig. 7:**
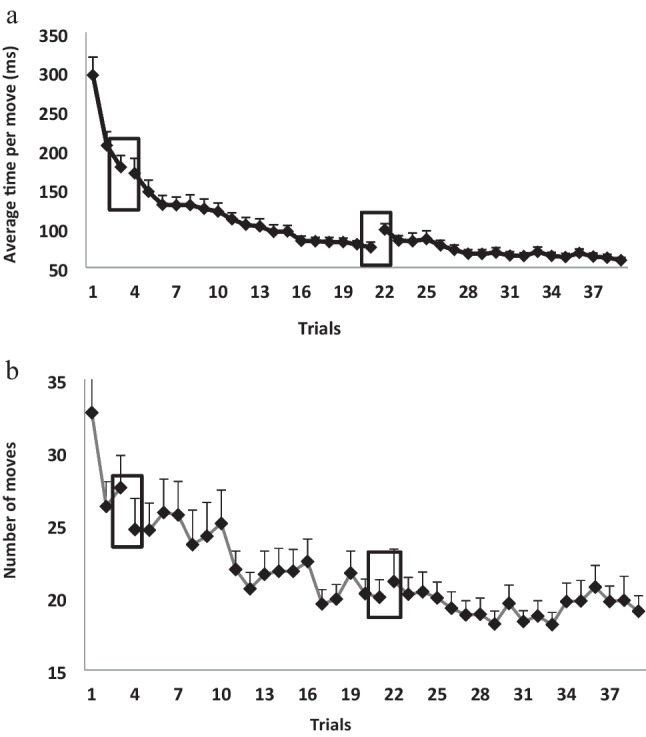
(**A**) The average time per move in each one of the trials. (**B**) The average number of moves, in the three sessions: session 1 (short training: blocks 1–3) and long training: session 2 (blocks 4–21) and session 3 (blocks 22–39), blocks, in the Tower of Hanoi puzzle (TOHP). Bars = *SE*

There was a significant learning trial effect, *F*(2, 28) = 22.09, *p* < 0.001, ${\eta }_{p}^{2}$ = 0.61, indicating a significant effect even after short training. There was also a significant effect for session, *F*(2, 38) = 77.52, *p* < 0.001, ${\eta }_{p}^{2}$ = 0.85, meaning there was a significant difference between performances in the three sessions. There was a significant interaction between learning trials and sessions, *F*(4, 56) = 14.19, *p* < 0.001, ${\eta }_{p}^{2}$ = 0.50, indicating that the level of learning changes differently in each of the learning phases; in the first session, the decrease was very steep. In session two the significant decrease continued, but more moderately, and by the third session the decrease was already very moderate (see Fig. 7a).

#### *Short training effect* – *average number of moves*

A significant learning trial effect was found, *F*(2, 28) = 4.85, *p* < 0.05, ${\eta }_{p}^{2}$ = 0.26. These results indicate a significant effect after short training. There was also a significant effect for session, *F*(2, 38) = 20.84, *p* < 0.001, ${\eta }_{p}^{2}$ =0.60, showing that there is a significant difference between performances in the three sessions. The interaction between learning trials and sessions was not statistically significant, *F*(4, 56) = 2.25, *p* = 0.075, ${\eta }_{p}^{2}$ = 0.14; the level of learning changes differently in each of the learning phases: in the first session there was a decrease in the number of moves, while in sessions 2 and 3 there were no additional decreases (stabilized) (see Fig. 7b).

#### *Long training effect* – *average time per move*

The results show a significant learning effect, *F*(17, 238) = 19.85, *p* < 0.001, ${\eta }_{p}^{2}$ =0.59. In addition there was a significant difference between the two sessions, *F*(1, 14) = 120.77, *p* < 0.001, ${\eta }_{p}^{2}$ = 0.90, so that performance was better in the third session. There was also a significant interaction between the sessions and trials, *F*(17, 238) = 4.30, *p* < 0.001, ${\eta }_{p}^{2}$ = 0.24, meaning that there is a difference between the sessions; the learning rate in session 2 is steeper than that in session 3 (see Fig. 7a).

#### *Long training effect* – *average number of moves*

A significant learning effect was found, *F*(17, 238) = 4.44, *p* < 0.001, ${\eta }_{p}^{2}$ =0.24, as well as a significant difference between the two sessions, *F*(1, 14) = 8.54, *p* < 0.05, ${\eta }_{p}^{2}$ = 0.38, so the performance is better in the third session. There was also a significant interaction between the sessions and trials, *F*(17, 238) = 3.67, *p* < 0.001, ${\eta }_{p}^{2}$ = 0.21, meaning that there is a difference between the sessions; the learning rate in session 2 is steeper than the rate in session 3 (see Fig. 7b).

#### *Delay effect* – *average time per moves*

**Fig. 8:**
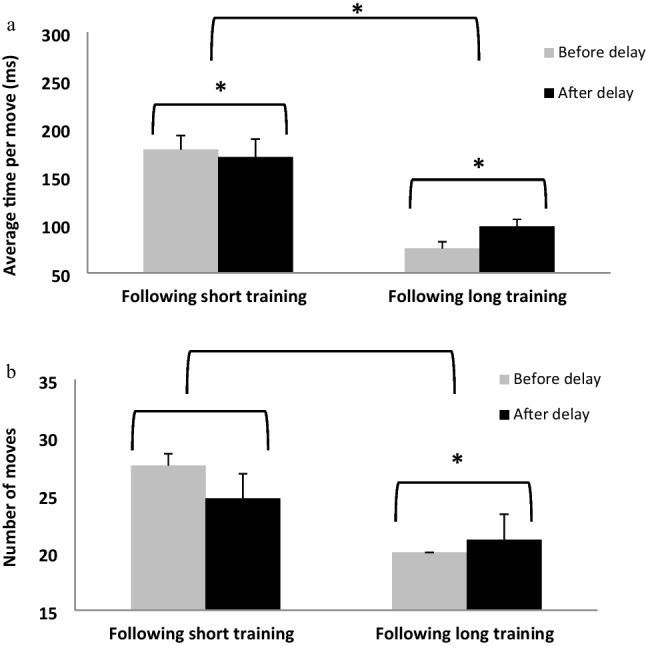
Comparison of the last block before the delay (light gray) to the first session after the delay (black) after a short training and also after long training in the Tower of Hanoi puzzle (TOHP) in the two measures: (**A**) The average time per move in each one of the trials. (**B**) The average number of moves in the TOHP. Bars = *SE*, * *p* < .001

There was no trial effect, *F*(1, 14) = 0.01, *p* = 0.92, ${\eta }_{p}^{2}$ = 0.001; however, there was a significant session effect, *F*(1, 14) = 40.44, *p* < 0.001, ${\eta }_{p}^{2}$ = 0.74. In addition, there was a significant interaction of session by trial, *F*(1, 14) = 17.03, *p* < 0.01, ${\eta }_{p}^{2}$ = 0.55. In a follow-up *t*-test analysis, results showed that there was a significant difference between the first and the second session, *t*(14) = 2.41, *p* < 0.05; the delay after the first phase, short training period, significantly improved performance level. There was also a significant difference between the last trial in session two and the first trial in session three, *t*(14) = -4.30, *p* < 0.005, however there was a reduction in performance level and not an improvement. The delay in the second phase of learning caused deterioration in performance (see Fig. 8a).

#### Delay effect - Average number of moves

The results showed there was no significant trial effect *F*(1, 14) = 0.29, *p* = 0.60, ${\eta }_{p}^{2}$ = 0.02, but there was a significant session effect, *F*(1, 14) = 31.70, *p* < 0.001, ${\eta }_{p}^{2}$ = 0.69. In addition, there was a significant interaction of session and trial, *F*(1, 14) = 9.98, *p* < 0.01, ${\eta }_{p}^{2}$ = 0.42**.** In a follow-up *t*-test analysis, results showed that there was no significant difference between the last trial in the first session and the first trial in the second session, *t*(14) = 1.19, *p* = 0.25. There was a significant difference between the last trial in session two and the first trial in session three, *t*(14) = -3.14, *p* < 0.05, but there was a reduction in performance level rather than an improvement. The delay in the second phase of learning caused a deterioration in the performance (see Fig. 8b).

The results of Experiment 2b show a significant improvement in performance in terms of average time per move and number of moves, after a delay that was afforded after a short training. Interestingly, after a delay post long training, there was a significant decline in performance in terms of average time per move and number of moves. These results indicate that in the performance of a cognitive task (TOHP), a consolidation effect was apparent after a short training (only for the average time per move, but not for the number of moves measure), in contrast to the motor task (FTSL), for which the consolidation effect was apparent only after long training.

In order to address the question whether the three TOHP trials had already entered the slow learning phase, which may explain the significant gains found after the first session, a follow-up experiment was conducted.

## Experiment 2c

### Methods

#### Participants

Participants (*n* = 20, 18 females) were undergraduate students at Bar-Ilan University (Israel) who took part in the experiment to fulfill academic requirements. Ages ranged from 20 to 27 years (*M* = 22.8 years).

#### Task and procedure

The cognitive task (TOHP) was as described in Experiment 1b. The experiment included two sessions, 24–48 h apart. In the first session, participants underwent a short training, consisting of just one trial, and 24–48 h later two more trials.

### Results

The two dependent measures were analyzed separately: average time per move and number of moves (as described in Experiment 1b). We compared the single trial in the first session to the first trial in the second session on the two measures.

#### Average time per move

The results showed that the difference between the single trial in the first session *(M* = 5.31, *SD* = 3.48) and the first trial in the second session *(M* = 3.93, *SD* = 2.41) was significant, *t*(19) = 3.45 *p* < .005, An additional *t*-test analysis was conducted, comparing the single trial in the first session to the average performance on trials 1 and 2 in session two. Results showed that the average time per move differed significantly from session 1 *(M* = 5.31, *SD* = 2.41) to session 2 *(M* = 3.35, *SD* = 1.42) *t*(19) = 3.57, *p* < .005.

#### Number of moves

The results showed that in terms of the number of moves, there was no significant difference between the single trial in the first session *(M* = 28, *SD* = 6.7) and the first trial in the second session, *(M* = 27, *SD* = 8.5) *t*(18) = 0.36, *p* = 0.72. An additional *t*- test analysis was conducted, comparing the single trial in the first session to the average performance of trials 1 and 2 in session two. The results showed that there was no significant difference between session 1, *(M* = 28, *SD* = 6.52) and session 2, *(M* = 27.52, *SD* = 10.67), *t*(19) = 0.21, *p* = 0.83. The results of Experiment 2c clearly show that for the average time per move even after a very short training of one trial, there were still significant gains in performance measures after the delay. These results reinforce the results of Experiment 2b, which showed consolidation effects after short training.

## General discussion

The aim of the current study was to examine the effect of a long delay of 24–48 h, an interval that could potentially accommodate memory consolidation processes – specifically the expression of delayed gains (post-session enhancement) in performance – following short versus long training in two types of tasks: a clearly specified movement sequence (FTSL) and a more cognitively challenging task – unspecified recursive-rule based movement sequence (TOHP). The first experiment was run to determine the amount of practice, in each of the two tasks, that would suffice for the trainees to attain a plateau in speed and accuracy of performance; attaining a plateau was suggested as a reliable indicator for initiating delayed gains (e.g., Hauptmann & Karni, 2002; Hauptmann et al., 2005); our work assumption was that the attainment of a plateau by the end of a training session can serve as an operational criterion for the attainment of a comparable phase of learning (skill acquisition) in two very different tasks.

It was expected that in both tasks, following short training, during the initial practice phase, when the task is relatively new, the delay interval (24- to 48-h long) would result in a (temporary) performance regression and, in any case, in no significant delayed gains in performance (Hauptmann et al., 2005; Karni, 1996; Korman et al., 2003). At the most, a continuation of the learning process was expected, in a manner similar to that occurring when no delay interval was afforded. In contrast, based on previous studies in a similar task (i.e., the finger-to-thumb opposition sequence learning task, e.g., Korman et al., 2003, 2007) it was expected that in the FTSL task, the 24- to 48-h long delay, if introduced after the participants have reached the performance plateau phase in the training session, would result in delayed gains in performance.

The question addressed here was whether, in the acquisition of the more cognitively complex task, the TOHP, delayed gains would emerge following the attainment of the plateau phase in task performance. From a theoretical framework two outcomes were considered in relation to the TOHP. One possibility was that if a common repertoire of basic neuro-behavioral processes of learning and memory underlies all forms of skill mastery (e.g., Karni, 1996), the expression of delayed gains was to be expected for participants practicing the TOHP, as in the FTSL. An alternative outcome was that the long delay interval would result in some loss of performance (forgetting) or at most a continuation of the learning process. This was based on the notion that differences are to be expected between the cognitive processes that underlie the learning of simpler versus conceptually more complex tasks (Moscovitch et al., 1993; Vakil & Hoffman, 2004). In complex tasks, even after a longer session, one may get a pattern of performance change other than that of delayed gains, perhaps more alike to the pattern observed after a delay interval introduced in early phases of practice in simpler tasks. An outcome other than the emergence of delayed gains could be expected if the amount of practice afforded before the long delay interval was not sufficient for the optimization of some of the (presumably many) subroutines comprising the complex task and/or their integration into a unitary-automatic task solution routine.

The acquisition of skill was expected to lead to contemporaneous improvements in both measures of performance, speed (RT, time-per-move, in the FTSL and TOHP, respectively) and accuracy (number of errors committed, number of moves, in the FTSL and TOHP, respectively), rather than a tradeoff between speed and accuracy (Chignell et al., 2014). Gains in both measures, specifically no speed-accuracy trade-off, is a reliable indication of acquisition of skill (Karni et al., 1995; Korman et al., 2003). Because in the FTSL task accuracy was maintained at ceiling level, the sensitive measure for performance gains, and the attainment of a plateau phase, was speed; in the TOHP both measures reflected the effects of training on performance and indicated an approach to plateau.

The current results show that robust learning occurred within the initial practice session in both tasks and, importantly, the course of this learning was characterized by the basic phases of skill acquisition – initially large gains from one-to-the-next task iteration (“fast” learning) but subsequently a clear trend to plateau, i.e., a process well modeled as a power function (Adi-japha et al., 2008; Vakil et al., 2014). Moreover, in both tasks, the affordance of the long-delay interval during the initial practice phase (i.e., following short training) resulted, on the whole, in a continuation of the learning trajectory initiated at the beginning of practice (the first practice session). The finding of shared patterns of performance gains, and the similar effects of the long delay, is in line with the notion of a common time-course characterizing skill acquisition across very different tasks. Nevertheless, there was a small, albeit temporary, loss of the gains in performance accrued the day before across the long-delay interval in the TOHP, but not in the FTSL, indicating a need for a quick re-setting of the task performance routine in the former, more complex, task, after the long-delay (Leizerowitz et al., 2023; Racsmány & Bencze, 2018); a reactivation or a warm-up trial that may be required in such a complex (not yet unitized) task.

It is reasonable to assume that at the beginning of a learning process, the learner uses the previously existing repertoire of task-solution modules and sub-routines to form a new task-solution routine (Karni, 1996; Telgen et al., 2014). As long as a new task-specific routine has not been finalized (such as during the “fast learning” phase), available (previously existing) routines that are relevant to task solution are tried out and adapted to the specific features and demands of the new task (e.g., Adi-japha et al., 2008; Karni, 1996; Karni et al., 1998; Olivier et al., 2022; Telgen et al., 2014). The current results show that in both the FTSL and the TOHP these adaptations can be maintained when an interval of 24–48 h (despite the potential interference from the participants’ daily activities) is introduced during the initial practice phase. Such maintenance of gains has been reported in previous studies, and ascribed to the use of a (previously) well-established routine in practicing the new task (Hockley et al., 2012; Verwey, 2003). However, we cannot rule out the possibility that more than maintenance occurs in the TOHP across the interval following short practice, because speed (time-per-move) improved. The TOHP is a demanding, complex task and is likely to require the engagement of presumably multiple routines, some that are familiar (key-presses on the keyboard) and some that are new (the recursive rule). It is likely that routines related to the recursive rule underlying the TOHP solution are not readily available for most individuals, and one cannot rule out the possibility that these new routines may be elaborated on and strengthened during the delay even after short practice. In addition, in a complex task such as the TOHP, the learner may need to rely more on declarative knowledge in order to establish the rule for solving the problem. Thus, it might be the case that after short training in the TOHP, in the delay interval (that included a night or two of sleep), new task-solution insights are elaborated on and strengthened (Wagner et al., 2004), leading to enhanced performance after the delay. The results therefore raise the possibility that delayed gains in performance may reflect different processes – the generation and consolidation of novel, optimized, task solution routines after long practice (e.g., Karni, 1996) and/or the emergence of new insights about how the task can be solved.

Our results, therefore, do not bear out our assumption that the attainment of a performance plateau by the end of a training session can serve as an operational criterion for the attainment of a comparable phase of learning (skill acquisition) in different tasks. The results of the TOHP indicate that the attainment of a plateau in performance by the end of a training session is not, by itself, a sufficient condition for the emergence of delayed gains in the performance of the practiced task. A possible explanation for the differential outcome of plateau attainment in the FTSL and TOHP may be that practice can lead to two different states-phases of stabilization of performance in the course of skill acquisition. Thus, *plateau* attainment can reflect a phase in task performance wherein the various available subroutines underlying the task are either at a temporary steady state – but a task-relevant set of subroutines has not been coordinated and finalized yet, or a state wherein a specific set of subroutines, from those available for the task, has been assembled and optimally integrated during practice and is undergoing in the following delay interval a process of consolidation (in the sense of an active improvement; Dudai et al., 2015).The latter state, which we conceptualize as an *asymptotic* state, constitutes the state wherein subsequent (delayed) performance gains can be achieved. In the asymptotic state, all or at least a critical set of the subroutines comprising a selected task solution set is made ready to be augmented by long-term synaptic plasticity, in the ensuing delay interval (Dudai et al., 2015; Karni, 1996). Thus, only after an asymptotic state has been attained, the subsequent latent, hours-long, process of consolidation will lead to the generation of a new, potentially performance-enhancing set of task-specific routines (Karni, 1996; Karni et al., 1998; Korman et al., 2003). From this perspective, delayed gains would emerge only after the attainment of an *asymptotic* state, as they constitute the behavioral expression of the consolidation process-dependent new neural machinery (Dudai et al., 2015). However, in the course of training, participants may attain, temporarily, states of stable performance – brief plateau states – wherein the conditions sufficient for the initiation of the processes subsequently expressed as delayed gains have not been fully met (yet). There are in fact indications for such temporary performance plateaus (often followed by bursts of increased performance variability) in the performance of individuals even in training on simpler tasks such as the FTSL task (Adi-japha et al., 2008).

The different pattern of results obtained for the FTSL and the TOHP may indicate a more general principle of the relationship between task complexity and the amount of practice required to attain and then consolidate a unitary task solution routine. Although consolidation processes can already be initiated for various aspects (sub-routines) of a complex task during the initial extended practice session (as suggested by the robust retention after the long practice session afforded for the TOHP in the current study), the expression of delayed, offline gains in task performance may be masked in such a complex task because performance reflects in parallel other task aspects that have not been optimized for task solution. Thus, the lack of a coherent unitary solution would be reflected in behavior as stabilization but not in the form of (measurable) delayed gains; delayed gains may be generated for some task aspects (sub-routines), but these effects would remain below threshold to be observable in overall task performance.

We would argue that the amount of training afforded in the extended practice session (in Experiment 2) on the relatively simple FTSL was sufficient for the participants not only to temporarily go through one or even more plateau phases but also reach an asymptotic state at the end of the session. In contrast, in the TOHP, perhaps due to the complexity of the task, the training afforded was sufficient to reach a plateau but not an asymptotic state.

Thus, the state attained in the TOHP after the 20 iterations of the task may be only a (temporary) plateau. This interpretation is supported by the finding that: (1) in the overnight session participants’ performance showed some regression and only after a few re-setting trials continued to show learning in terms of time-per-move, and (2) by the large fluctuations in the number of moves to solution after the delay. We propose that both measures of performance provide an indication that a specific set of subroutines has not yet been stabilized nor optimally integrated by the end of the first session.

The proposed framework has testable predictions. One prediction is that given further practice sessions the performance in the TOHP would eventually reach the asymptotic state, and this will subsequently open the way to the expression of delayed gains. We can assume that the specific amount of training required to attain an asymptotic state is perhaps contingent on the complexity of the task, but we do not have an a priori operational indicator for when the necessary amount of training (task iterations) for an asymptotic state has been attained online; currently the putative indicator is post hoc the expression of delayed gains. The challenge thus is to discover an indicator for the asymptotic state. One possible approach may be to capitalize on individual performance variability measures as indicators of transitions from one phase of learning to another (Adi-japha et al., 2008). Another possibility would be to take a neuroimaging approach. The transition from controlled to automatic processing can be reflected in changes in brain activity shifts within cortical areas and sub-cortical areas (such as the basal ganglia) (e.g., Pinsard et al., 2019; Schneider & Chein, 2003). Thus, changes in brain activation may indicate the nature of the asymptotic state attained at the end of a skill practice session.

It should be noted that although in the current study the TOHP task is referred to as a complex conceptual task, and the FTSL is referred to as a simple motor task, we do not propose that motor or perceptual tasks cannot be complex or that conceptual tasks cannot be simple; i.e., simple and complex are not synonymous with perceptual-motor and conceptual task demands, respectively. Based on our findings we propose that in simple tasks it may be possible to obtain a new skilled routine even after a single session of practice, if sufficient practice is afforded to enable the attainment of a plateau phase, and this routine can be subsequently consolidated during a long delay interval (and improved upon if more practice sessions are subsequently afforded). In complex tasks, as the current findings indicate for the TOHP, the amount of practice that allows for the attainment of a plateau phase within the practice session may suffice to enable only a stabilization of the newly acquired skill in task performance. These differential outcomes suggest the possibility that the attainment of a plateau phase within a practice session does not necessarily indicate that a comprehensive task solution routine has been attained in the trained task. We propose, therefore, that the stabilization of performance gains within a practice session – the attainment of a plateau phase – can indicate one of two states: (1) the attainment of a comprehensive task solution routine (as suggested e.g., Karni et al., 1998), and (2) a preservation of work-in-progress on a (yet unfinished) task solution routine. However, the performance level after a long post-practice interval can differentiate these two states. The expression of delayed gains across a post-practice delay interval can be viewed as a behavioral marker of the establishment of a (new) comprehensive task solution routine, while the stabilization of performance gains across the delay interval reflects a preservation of the work-in-progress gains towards the to-be-continued process of generating a comprehensive task solution routine.

Going somewhat beyond the data, this notion could have potential implications for education, rehabilitation, or professional training. In everyday life we ​​are often required to learn both simple routines (such the code for the car ignition or a house alarm system) and more complex routines (such as mastering a new computer program or complex equipment). The current results imply that even when it appears that, following a practice session when proper task execution has been achieved and the individual’s best (asymptotic) performance attained, this does not necessarily indicate lasting proficiency, specifically not in complex skills. In order to attain stabilization and lasting skill mastery, practice may need to be continued despite the attainment of a performance plateau (e.g., Karni, 1996). For example, when practice should be considered sufficient when children learn to solve mathematical problems at school cannot be based on the attainment of “best” performance (Hatano & Inagaki, 1986).

In conclusion, the results of the current study show the intricate nature of the processes underlying skill learning and the generation of long-term “how to” memory. An important insight brought to the forefront by the findings of the current study is that the amount of training required to attain delayed “offline” gains in performance of a newly leaned task may be contingent on the nature of the task and the learner’s prior experience (e.g.,Olivier et al., 2022; Schwizer Ashkenazi et al., 2022). Despite a characteristic time-course for skill acquisition in tasks of different domains and complexity (e.g., a power function like learning curve), the behavioral outcome of practice may diverge in the more advanced stages of skill acquisition and specifically in the ability to express off-line consolidation phase gains. The current results suggest that a similar pattern of behavioral changes during learning may not indicate a similar phase in the learning process. Thus, caution is called for when comparing and interpreting the results from studies that use diverse tasks (in terms of complexity) and procedures (e.g., the amount of practice afforded); one cannot assume that in training a given “task” one necessarily taps into the same processes of learning and memory processes.

## Data Availability

Data and material are available upon request from the corresponding author (EV).

## Declarations

### Authors’ Note

This study was carried out as part of a PhD dissertation by Iris Lowenscuss-Erlich Bar-Ilan University, Ramat Gan, Israel, under the supervision of EV and at AK.
